# A mathematical model as a tool to identify microRNAs with highest impact on transcriptome changes

**DOI:** 10.1186/s12864-019-5464-0

**Published:** 2019-02-06

**Authors:** Marzena Mura, Roman Jaksik, Anna Lalik, Krzysztof Biernacki, Marek Kimmel, Joanna Rzeszowska-Wolny, Krzysztof Fujarewicz

**Affiliations:** 1 Department of Systems Engineering, Institute of Automatic Control, Silesian University of Technology, ul. Akademicka 16, 44-100 Gliwice, Poland; 2 Ardigen S.A., ul. Bobrzyńskiego 14, 30-348, Cracow, Poland; 3 Centre of Biotechnology, Silesian University of Technology, ul. Bolesława Krzywoustego 8, 44-100 Gliwice, Poland; 4 Departments of Statistics and Bioengineering, Rice University, MS 138, 6100 Main, Houston, TX 77005 USA; 5 Department of Medical and Molecular Biology, School of Medicine with the Division of Dentistry in Zabrze, Medical University of Silesia in Katowice, Katowice, USA

**Keywords:** miRNA, miRNA-mRNA interactions, ionizing radiation, stressing factor

## Abstract

**Background:**

Rapid changes in the expression of many messenger RNA (mRNA) species follow exposure of cells to ionizing radiation. One of the hypothetical mechanisms of this response may include microRNA (miRNA) regulation, since the amounts of miRNAs in cells also vary upon irradiation. To address this possibility, we designed experiments using cancer-derived cell lines transfected with luciferase reporter gene containing sequences targeted by different miRNA species in its 3′- untranslated region. We focus on the early time-course response (1 h past irradiation) to eliminate secondary mRNA expression waves.

**Results:**

Experiments revealed that the irradiation-induced changes in the mRNA expression depend on the miRNAs which interact with mRNA. To identify the strongest interactions, we propose a mathematical model which predicts the mRNA fold expression changes, caused by perturbation of microRNA-mRNA interactions. Model was applied to experimental data including various cell lines, irradiation doses and observation times, both ours and literature-based. Comparison of modelled and experimental mRNA expression levels given miRNA level changes allows estimating how many and which miRNAs play a significant role in transcriptome response to stress conditions in different cell types. As an example, in the human melanoma cell line the comparison suggests that, globally, a major part of the irradiation-induced changes of mRNA expression can be explained by perturbed miRNA-mRNA interactions. A subset of about 30 out of a few hundred miRNAs expressed in these cells appears to account for the changes. These miRNAs play crucial roles in regulatory mechanisms observed after irradiation. In addition, these miRNAs have a higher average content of GC and a higher number of targeted transcripts, and many have been reported to play a role in the development of cancer.

**Conclusions:**

Our proposed mathematical modeling approach may be used to identify miRNAs which participate in responses of cells to ionizing radiation, and other stress factors such as extremes of temperature, exposure to toxins, and drugs.

**Electronic supplementary material:**

The online version of this article (10.1186/s12864-019-5464-0) contains supplementary material, which is available to authorized users.

## Background

Response of human cells to electromagnetic radiation is of paramount importance in many contexts, including the intertwined fields of carcinogenesis and radiation therapy of cancer. Obtaining insights into the qualitative and quantitative nature of the response is complicated by superposition of potential mechanisms of primary and secondary response. For this reason, we focus on the short-term transcriptional response (mostly up to 1 h past irradiation), and in particular its regulation by other RNA species. Because of this relatively narrow focus, it seems possible to propose that a simple, non-mechanistic, statistical model helps to identify and quantitate the leading components of this regulation and that the modelling outcomes are free of statistical bias.

Rapid changes in the expression of many messenger RNAs (mRNAs) follow exposure of cells to irradiation by X-rays, and the change of expression of up- or down-regulated mRNAs is strongly correlated with the distribution of microRNA (miRNA) recognition motifs in their non-coding 3’-UTR sequence [1]. One of the factors regulating the expression of mRNAs is degradation of transcripts mediated by miRNAs, small noncoding RNAs strongly conserved throughout evolution which, together with the proteins from the Argonaute family, function as RNA-induced silencing complexes (RISCs) [2–4]. Complementarity of a 6–8 nucleotide-long region at the 5′ end of a miRNA (the “seed” region) to a target sequence in a primary transcript is necessary for mRNA-RISC interaction and leads to faster or slower mRNA degradation [5]. A single miRNA may regulate many mRNAs and a single transcript may contain sequence motifs targeted by different miRNAs [5]. The miRNA-directed regulation plays an important role in establishing the expressions of mRNAs and their translation rates and is involved in cell proliferation, differentiation, apoptosis, stress responses, immune responses, and diseases including cancer [6–10]. It is therefore important to understand the molecular mechanisms by which miRNAs regulate mRNA levels and how the regulation is affected by stress factors.

**Fig. 1:**
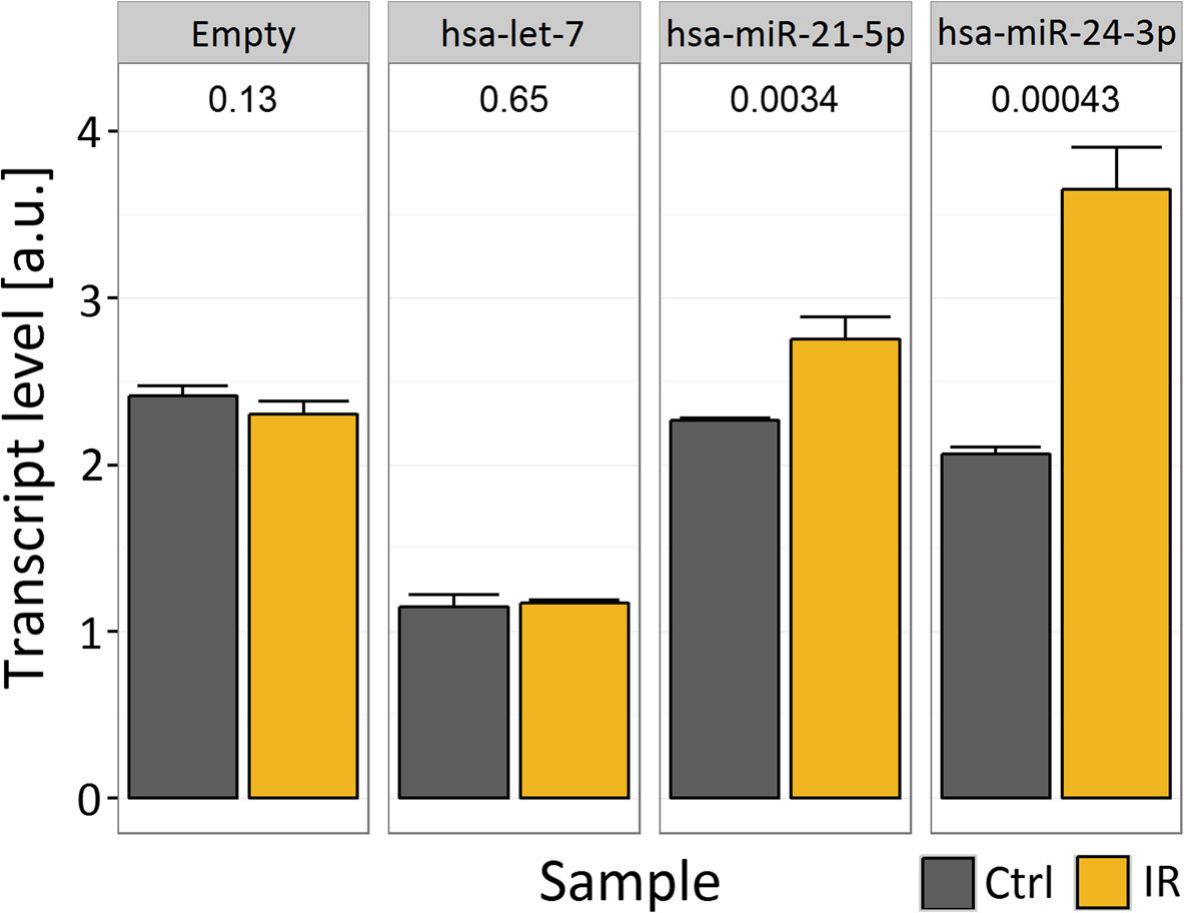
Levels of Renilla luciferase mRNA in control and irradiated Me45 cells. Cells were transfected with a reporter gene plasmid containing a Renilla luciferase gene with the same promoter and coding sequence but whose primary transcript contained target sequences for different miRNAs versus those that did not contain the targets (empty). Renilla mRNA was assayed by RT-PCR and normalized to the Firefly mRNA to exclude differences in transfection efficiency between experiments. Error bars represent standard deviations of values obtained in three experiments, and *p*-values show the significance of differences between control and irradiated samples tested by a two sample t-test

The motivation for research described in this paper was provided by preliminary experimental studies, in which we examined the influence of motifs in the 3’ UTR of a luciferase reporter gene (see Methods), recognized by members of let-7 family, miR-21, and miR-24, on ionizing radiation-induced changes in the luciferase mRNA expression. We were mostly interested in early-phase response, before any later effects of irradiation might interfere. Therefore, we focused on the change in luciferase expression between the 0 h (immediately before irradiation), and the 1 h time points. A statistically significant increase (*p*-value < 0.05) of expression of luciferase mRNA occurred after irradiation when the primary transcript contained motifs targeted by miR-21 or miR-24, but in contrast no significant increase occurred in transcripts targeted by let-7 or devoid of miRNA-targeted motifs (Fig. 1). This suggested that mRNA response to irradiation depended on its interactions with specific miRNAs. However, to understand the process in quantitative detail and build a mathematical model, we decided to employ microarrays, which deliver data on multiple mRNAs and miRNA.

The processes of regulation of gene expression by miRNAs are subject of a number of studies using systems biology approaches [11, 12]. They were also modelled using approaches ranging from a single mechanism [13, 14] to multiple mechanisms influenced by miRNA action [15]. Modelling of these processes is based on data from miRNA and mRNA microarrays [16–23] or RNA sequencing [24], which allow tracking changes in the level of multiple miRNAs and mRNA. Large-scale studies have been based on measurement of correlations [16, 19, 20], linear regression [17, 25], partial least square regression with bootstrap-based statistical tests [18], least angle regression [23], chemical kinetics eqs. [21], or Bayesian methods [26]. Methods to find relevant regulatory associations are based on expression data for miRNAs and mRNAs and publicly-available miRNA target prediction algorithms such as MAGIA [27] and MIMA [28]. The method presented in this paper is based on dynamic changes of mRNA expression, as opposed to previous studies that mostly considered single time point measurements (see the summary of the most popular methods in ref. [29]).

In this paper we propose a simple mathematical model constructed based on microarray data. The purpose of the model is to predict changes of mRNA levels in cells exposed to ionizing radiation which may contribute to identifying the miRNAs and mRNAs involved. The hypothesis tested by the model is based on our previous studies of the changes of mRNA and miRNA levels in cells after exposure to X-radiation [1, 30], which suggests that in many cases changes in mRNA expressions result from radiation-induced perturbation of the interactions of miRNAs with mRNAs. We propose that the influence of cognate miRNAs on the change of level of single mRNA depends on the number of miRNA binding sites in the 3′-untranslated region (UTR). The mechanism of perturbations in miRNA-mRNA interactions is not specifically included in the model, which therefore might be classified as statistical. Speculatively, the mechanism may be the consequence of RNA or RISC proteins damage by radiation or changes in miRNA biogenesis and concentration. Changes of mRNA levels computed from the model show good correlation with experimental microarray data for irradiated cells. This suggests that in some cell types major part of these changes can be assigned to perturbed mRNA-miRNA interactions and that only a small subset of miRNAs (7% of miRNAs expressed in melanoma Me45 cells) participates in this effect.

## Methods

### Cell culture and irradiation

Human melanoma Me45 cell line (established in the Center of Oncology in Gliwice), K562 cell line, and two HCT116 cell lines with different P53 status (ACCT collection) were grown in DMEM/F12 medium in RPMI 1640, both with L-glutamine (Sigma-Aldrich), 10% fetal bovine serum (Gibco), and 80 μg/ml gentamycin at 37 °C in a humidified atmosphere and 5% CO_2_. Exponentially growing cells were irradiated at room temperature with 4 Gy (1 Gy/min) of 6 MV X-ray photons generated by a therapeutic accelerator (Clinac 600) in fresh culture medium (changed 15 min before irradiation).

### Transfection with reporter genes

Me45 cells were transfected with psiCHECK2 plasmid (Promega) containing two luciferase genes, *Firefly* luciferase being a reference and *Renilla* luciferase with eight tandem repeats of various miRNA target sites in its 3’UTR being the reporter gene. The plasmid contained sequences targeted by either the miRNA let-7, miR-21 or miR-24. The let-7 target sequence had motif TCGAGACTATACAAGGATCTACCTCAG, 71.75% average complementarity to the target sequences of various mature let-7 family members [31]. The miR-21 target sequence had motif TCAACATCAGTCTGATAAGCTAAA, 100% complementarity to the mature miR-21 target; the two last AA form a spacer to limit complementarity for nonspecific binding. The miR-24 target sequence had motif ATACGACTGGTGAACTGAGCCG, 68% complementarity to the mature miR-24 target. Sequence synthesis, insertion, and verification were performed by the BLIRT S.A. company (Gdansk, Poland). The unmodified plasmid was used as a control (empty). Transfection was performed with lipofectamine (Invitrogen) according to the supplier’s protocol. Transfected cells were irradiated in the same conditions as non-transfected cells.

### Measurement of mRNA and miRNA levels

Microarray data for normal cell lines: AG1522, PBMC, HCAEC, and cancer cell lines: DU145, SC3, MOLT4 analyzed in this paper were downloaded from ArrayExpress database [32]. Additional file 1: Table S1 includes ID numbers of individual experiments.

mRNA and miRNA levels were estimated by Affymetrix (Human Genome U133A) and Agilent (G4870A SurePrint G3 Human v16 miRNA 8x60k) microarrays 1, 12 and 24 h after irradiating the cells, with control being the non-irradiated cells at 1 h, as described in [30]; these data are available in the ArrayExpress database (accession numbers E-MEXP-2623, and E-MTAB-5197 for mRNAs and miRNAs, respectively). The mRNA and miRNA datasets for Me45, K562 and HCT116^+/+^ cell lines were already explored. In the previous publications authors focused on the relation between reactive oxygen species and miRNA [1] and bystander effects [30, 33]. HCT116^−/−^ expression levels were not published previously. All datasets were normalized by the standard RMA method [34]. Only mRNAs and miRNAs with expression above the noise level were selected for analysis using the GaMRed software, which uses Gaussian mixture models [35]. The default parameter values were applied, i.e., number of initial conditions 100, number of components 2 to 8, Expectation-Maximization (EM) algorithm precision level 10^−2^, and maximum number of EM interactions 5000. Two methods were used for choosing the number of components to remove, the “top three” rule which finds groups of genes with high, medium or low expression and removes others, or the “*k*-means” method which uses *k*-means clustering and statistical information to remove clusters of non-informative genes [35]. Both methods returned the same noise level threshold. The noise level threshold does not have a significant impact on the prediction method; a detailed analysis is presented in Supplementary Material (Additional file 2: Figure S1). The numbers of mRNAs and miRNAs considered in individual cell lines are presented in Additional file 3: Table S2.

### Identifying miRNA-targeted motifs in primary transcripts

miRNA directly binds to a subset of mRNAs in binding site regions. The numbers of these sites are model parameters (see further on). Several tools for miRNA target prediction have been developed using different approaches [36]; we used information from four different algorithms. The number of miRNA binding sites was estimated using:miRanda3.3a [37], TargetScan [38], RNAhybrid [39], and NucleoSeq [40]. As different algorithms may return different results, the influence of a particular *i*-th miRNA on the *j*-th mRNA was expressed as the weighted sum of all four algorithms:

$$ c{\prime}_{ji}=\sum \limits_{k=1}^a{c}_{ji}^k{w}_k $$

where *a* is the number of algorithms used (*a* = 4 here), ${c}_{ji}^k$ is the number of binding sites for the *i*-th miRNA in the 3’UTR of the *j-*th mRNA predicted by the *k*-th algorithm, and *w*_*k*_ is the weight assigned to the *k*-th algorithm. The weight coefficient values for each method were allocated in proportion to the number of the features considered by a given algorithm. Table 1 presents prediction features used by the 4 algorithms [36].

**Table 1:** Algorithms for predicting miRNA target sequences

Method	Text scan for miRNA motif	Binding energy	Sequence conservation	RNA folding	Weight coefficient
miRanda3.3a	Yes	Yes	No	Yes	0.3
TargetScan	Yes	Yes	Yes	No	0.3
RNAhybrid	Yes	Yes	No	Yes	0.3
NucleoSeq	Yes	No	No	No	0.1

Additional settings used in prediction algorithms include: *strict* option of the miRanda which requires strict alignment with the seed region and *p*-value of < 0.05 and a free energy ≤ − 30 for predicted miRNA-mRNA hybrid binding sites.

### Identification of sequence motifs which potentially influence mRNA levels

Additional information, not included directly in the model but useful for interpretation of results, concerned mRNA properties. Adenylate-uridylate-rich (ARE) motifs were identified using NucleoSeq [40] based on 3′-untranslated regions (UTRs) from the RefSeq transcript database and the TTATTTAWW consensus sequence [41]. Messenger RNA turnover times were derived from the experiments of Tani et al. on HeLa cells [42]. The association of transcription factor (TF) response elements with genes was based on a ChIP-Seq experiment performed by the ENCODE project [43]; a TF-gene association table was created using a map of TF-binding site positions in the genome available as a USCS track (wgEncodeRegTfbsClusteredV3). A TF was assumed to regulate a particular gene if its binding site was located between 5000 bp upstream and 1000 bp downstream of the transcription start site, taking into account the strand directionality of the gene.

### A model to predict changes of mRNA levels in irradiated cells

**Fig. 2:**
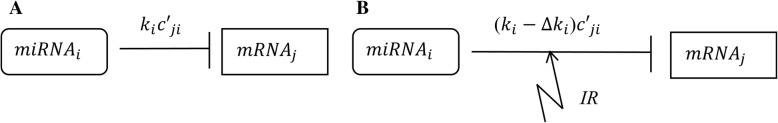
Negative impact of the *i*-th miRNA on the expression level of the *j*-th mRNA (**a**) in normal conditions or (**b**) in irradiated cells

We assumed that the change of expression of a mRNA in irradiated cells depends on the interacting miRNAs, and we considered that this principle could be applied generally to the expressions of mRNAs in a population of cells at cell cycle equilibrium. Interaction of a RISC complex with a transcript depends on the formation of a miRNA-mRNA hybrid, whose probability is determined predominantly by the number of miRNA-targeted motifs in the transcript and by the availability of the cognate targeting miRNAs. The impact of a particular (*i*-th) miRNA on the level of a particular (*j*-th) mRNA, shown schematically in Fig. 2a, is described by the expression:

1 $$ -{k}_i{c}_{ji}^{\prime }{miRNA}_i $$

where *k*_*i*_ is a proportionality factor (specific for the *i*-th miRNA and reflecting its activity), *miRNA*_*i*_ is the log_2_ transformed expressions of the *i-*th miRNA, and *c'*_*ji*_ is coefficient reflecting the number of motifs recognized by a particular (*i*-th) miRNA on a particular (*j*-th) transcript (identification of the number of miRNA-targeted motifs *c'*_*ji*_ for specific cellular mRNAs is described in Methods). The minus sign in Eq. (1) denotes the negative impact of interaction with a transcript. Eq. (1) represents effect of miRNA inducible gene silencing in unperturbed cellular conditions.

Our model proposes that in irradiated cells, the outcome (Equation (1)) is modified by decreasing the proportionality factor *k*_*i*_ by *∆k*_*i*_, depending on a change of the miRNA-mRNA interactions, as shown in Fig. 2b and expressed mathematically by the formula:

2 $$ -\left({k}_i-\Delta  {k}_i\right)c{\prime}_{ji}{miRNA}_i $$

In other words, the effect of miRNA-inducible gene silencing can change after cell irradiation. Interpretation of the notation used in Equation (2):

- if *∆k*_*i*_ > 0, then the negative regulation *decreases* so that expression of the *mRNA*_*j*_*increases*;
- if *∆k*_*i*_ < 0. then the negative regulation *increases* so that expression of the *mRNA*_*j*_*decreases*.

Subtracting Equations (1) and (2) side-by-side, we obtain the change of the outcome resulting from irradiation:

3 $$ \Delta  {k}_ic{\prime}_{ji}{miRNA}_i $$

Most transcripts contain multiple recognition motifs for one or different miRNAs [44]. In a parsimonious form, the influence of irradiation for a particular mRNA can be estimated by the sum of the influences of different miRNAs:

4 $$ \sum \limits_{i=1}^{N_{mi}}{\Delta  k}_ic{\prime}_{ji}{ mi RNA}_i $$

where *N*_*mi*_ is the number of types of miRNA which recognize the transcript. The total effect of irradiation on the level of a *mRNA*_*j*_, expressed as a binary logarithmic fold change (FC), can be described by the expression

5 $$ {FCmRNA}_j=\sum \limits_{i=1}^{N_{mi}}\Delta  {k}_ic{\prime}_{ji}{ mi RNA}_i+{b}_0 $$

which is a prediction of the experimentally observed fold change

6 $$ FCmRN{A_j}^{exp}={mRNA}_j^{IR}-{mRNA}_j^0 $$

where ${mRNA}_j^0$ and ${mRNA}_j^{IR}$ are the log_2_ transformed expressions of the *j*-th mRNA before and after irradiation. The additional constant *b*_0_ equation (5) represents possible change of the expression of a mRNA which are independent of miRNAs and are similar for all mRNAs, for example breakage of a fraction of mRNA molecules by radiation. Usually *b*_0_ has order of magnitude of 10^− 1^.

The *c*_*ji*_ elements for all mRNAs and all miRNAs form a matrix *C* of size *N*_*m*_ × *N*_*mi*_, where *N*_*m*_ is the number of different mRNAs and *N*_*mi*_ is the number of different miRNAs. Each row of the matrix *C* created for a particular gene sums the information on the impact of different miRNA-mRNA interactions on the expressions of one particular mRNA. This model can be written for all mRNAs as a matrix equation:

7 $$ FCmRNA=A\Delta  k+b $$

where:

$$ {\displaystyle \begin{array}{c}A={C}^{\prime}\mathit{\operatorname{diag}}(miRNA).\\ {} FCmRNA={\left[{FCmRNA}_1,{FCmRNA}_2,\dots, {FCmRNA}_{N_{mi}}\right]}^T\\ {}\begin{array}{c}\Delta  k={\left[{\Delta  k}_1,{\Delta  k}_2,\dots, {\Delta  k}_{N_{mi}}\right]}^T\\ {}\begin{array}{c} miRNA={\left[{ mi RNA}_1,{ mi RNA}_2,\dots, { mi RNA}_{N_{mi}}\right]}^T\\ {}b={\left[{b}_0,{b}_0,\dots, {b}_0\right]}^T\end{array}\end{array}\end{array}} $$

**Fig. 3:**
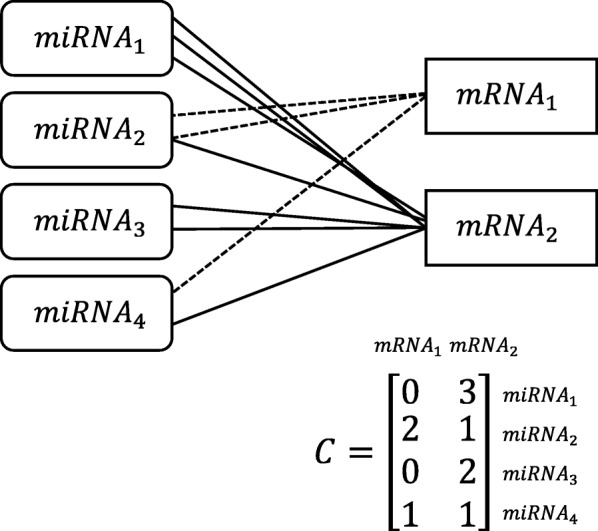
Design of matrix *C*. Each line joining a mRNA and a miRNA symbolizes the interaction of a miRNA with a target motif in a transcript (one line is one interaction, dashed line for miRNA1, solid line for mRNA2). Coefficients *c*_*ji*_ are obtained based on miRanda3.3a [34], TargetScan [35], RNAhybrid [36], and NucleoSeq [37] algorithms (Table 1)

Elements *A*_*ji*_ of matrix *A* are equal to the impacts of *miRNA*_*i*_ on *mRNA*_*j*_. Matrix *A* is the product of matrix *C*^’^ composed of elements reflecting the numbers of motifs recognized by different miRNAs (a simple example is shown in Fig. 3) and a diagonal matrix reflecting the concentration of these miRNAs. The matrix was validated by comparison with 3 differently randomized matrices: (Additional file 4: Figure S2): (1) simulated matrix *C* with numbers drawn from the uniform distribution from the [0, 1] interval, (2) permuted databases-based *C* matrix, (3) permuted *C* matrix with model parameters estimated based on the original *C* matrix. This model is simple, but it is useful only if the values of parameter *∆k* are known, otherwise the mRNA fold change values cannot be calculated.

Matrix Equation (7), after substituting in the left side the experimentally observed fold changes of mRNA expressions (*FCmRNA*^*exp*^), constitutes a set of *N*_*m*_ equations with *N*_*mi*_ unknown coefficients *∆k*_*i*_. There are more equations than coefficients and they are algebraically inconsistent because of the limited accuracy of microarray estimation of mRNA and miRNA levels and the fact that not all mRNA expressions are regulated by miRNA. On the other hand, Equations (7) are linear with respect to the coefficients Δ*k*_*i*_, which therefore can be estimated using the Least Squares Method. The calculated Δ*k*_*i*_ values for all miRNAs expressed in Me45, K562, HCT116^+/+^ and HCT116^−/−^ cells together with other characteristics of the miRNAs are shown in Additional file 5: Table S3.

## Results

### Comparing model simulations to experimental data - validation of the model

**Fig. 4:**
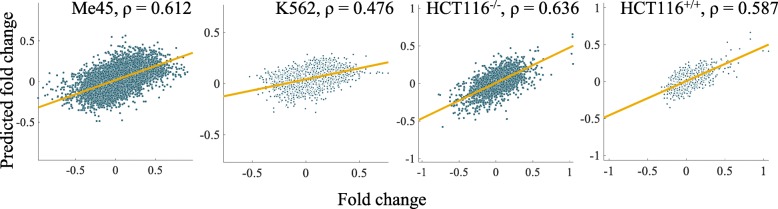
Scatterplots demonstrating correlations between the predicted and the experimentally observed fold changes of mRNA levels in four cell lines experimentally studied by us

Estimated values of parameters *∆k*_*i*_ were used in the model to predict the irradiation-induced mRNA changes and to compare the model calculations to the experimentally measured fold changes of mRNA expressions after irradiation in Me45, K562, HCT116^+/+^ and HCT116^−/−^ cells (Fig. 4). The Spearman’s correlation coefficient between the predicted and experiment-based fold changes of mRNAs was 0.612 for Me45 cells. It indicates that the miRNA-based regulation assumed in the model has a significant impact on the post-irradiation mRNA profiles. The components of the parameter vector *∆k*, which characterize the influence of particular miRNAs on the change of the expression of a mRNA, differ among cell lines. This suggests that *∆k* and the predicted results may be influenced by differences in the sets and properties of expressed mRNAs among these cell lines. For example, the motifs targeted by a particular miRNA could vary or have different accessibility in different cell lines..

**Fig. 5:**
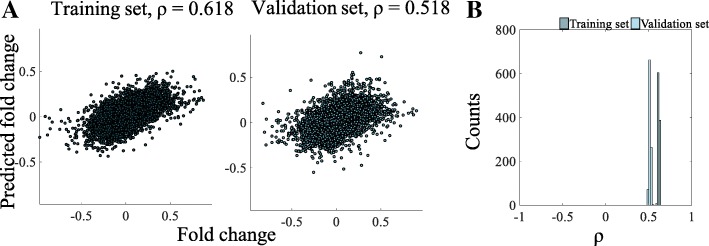
Model validation for the Me45 cell data. The mRNA dataset was randomly split into the training and validation set and parameters ∆k were estimated based on the training set, and then applied to generate model predictions based on the validation subset. Depicted are the scatterplots of empirical vs. predicted mRNA fold changes in training (**a**) and validation (**b**) datasets, and the histograms of the corresponding correlation coefficients (**c**) based on 10,000 random splits

To estimate the influence of the mRNA profile on parameter vector *∆k* and the quality of model predictions, we divided the dataset of mRNA expressions into two random subsets. These are called the training set and validation set. Parameters estimated using the training set were then used to predict the miRNA-dependent mRNA expressions using the validation set. Resulting miRNA-mRNA scatterplots and histograms are depicted in Fig. 5 (Me45 cell line) and in Additional file 5: Table S3 (other cell lines). Scatter plots show the relation between experimental mRNA fold change values and those resulting from modelling for one example of training and validation set. The values of correlation coefficients are high: 0.618 and 0.518, for the training and validation sets correspondingly. Similar values were obtained for 1000 random datasets, as shown in the histogram.

In the Affymetrix microarrays a significant proportion of transcripts has been present more than once (43% in Me45 cell line, Additional file 6: Figure S3). Since the multiple readings of transcripts of the same gene have highly correlated expression levels, this may result in inflation of the empirical correlation coefficient. Therefore, for the final analysis (Fig. 5 and Additional file 3: Table S2) the superfluous expression levels have been removed. The adjusted (based on genes) and raw (based on transcripts) correlation coefficients are depicted in Additional file 3: Table S2.

As described in the Methods, we also ran our model on different datasets available in public databases including a variety of normal and cancer-related cell lines (see Methods for detail). Cells were irradiated with different ionizing radiation doses and the transcriptomes were assayed at different times after irradiation (see in Additional file 7: Figure S4 and Additional file 3: Table S2). Additional file 3: Table S2 summarizes properties of each dataset (number of expressed mRNAs and miRNAs), experimental conditions (radiation dose in Gy, time of observation) and results of model simulations described as Spearman correlation coefficient values.

### Different groups of miRNAs have positive or negative effects on mRNA expressions in irradiated cells

The sign of parameter *∆k*_*i*_ indicates increase (−) or correspondingly decrease (+) amplitude of negative control of the expression of a corresponding mRNA by the *i*-th miRNA. The relationship between *∆k*_*i*_’s sign and the structure of a miRNA may provide insights into modifications of miRNAs which occur after irradiation of cells. We classified all miRNAs into two subgroups based on the sign of parameter *∆k*_*i*_ and compared their features, such as length, GC content, number of unpaired bases in the secondary structure of the corresponding pre-miRNA, and other features (see Additional file 5: Table S3 in Supplementary Material). We then performed Mann–Whitney U tests to check if the two samples originate from the same population. Table 2 compares the average values of some of these features calculated for all miRNAs and the subgroups characterized by negative or positive values of *∆k*_*i*_. Cell line Me45 is used as the reference since statistically significant differences have been found only for this cell line. In general, miRNAs in the *∆k*_*i*_-positive subgroup targeted a larger number of transcripts but had lower expression levels than miRNAs in the *∆k*_*i*_-negative subgroup. However, most features had similar values in both subgroups and in most cases, the differences between the subgroups were not statistically significant. The exceptions are the GC content in miRNA (*p*-value 0.003) and the seed motif (*p*-value 0.02).

**Table 2:** Features of miRNA subgroups with negative or positive *∆k*_*i*_ , with Me45 cells as reference

Features	Me45 cells			Concordance in other cell lines		
	Average over miRNAs with positive *∆k*_*i*_	Average over all miRNAs	Average over miRNAs with negative *∆k*_*i*_	K526	HCT116^+/+^	HCT116^−/−^
Length of pre-miRNA	86.02	86.55	87.18	+	–	–
Hairpin length	45.72	46.00	46.33	+	–	–
Length of mature miRNA	21.66	21.61	21.56	+	+	–
GC content in miRNA	**54.59** ^**b**^	52.60	**50.24** ^**b**^	+	–	+
GC content in seed motif	**56.06** ^**b**^	53.93	**51.40** ^**b**^	+	–	+
Number of targeted transcripts ^a^	3807.92	3711.41	3597.03	+	+	+
Expression level (logarithmic)	4.43	4.52	4.63	+	+	+

^a^Average of values calculated for each single miRNA, based on targeted motifs obtained as described in Methods

^b^Boldface numbers indicate statistically significant differences

Plus sign indicates concordance (i.e., sign of the difference of feature averaged over miRNAs with positive and negative *∆k*_*i*_, the same as in Me45 cells); minus sign – lack of concordance

### Particular miRNAs are significant for changes of mRNA expressions after irradiation

**Fig. 6:**
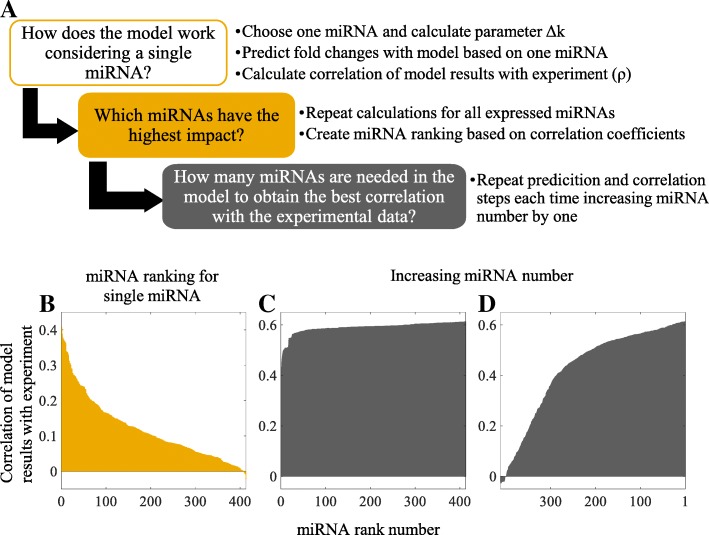
Influence of individual miRNAs on the prediction of radiation-induced changes of mRNA levels in Me45 cells. **a** Steps in establishing the significance of particular miRNAs in radiation-induced changes of mRNA levels. **b** Ranking miRNA according to correlation coefficient from highest to lowest. **c** Using an increasing number of miRNAs added ordered by decreasing rank. **d** Using an increasing number of miRNAs added ordered by increasing rank

We decided to examine if all miRNAs had the same impact on changes of the global mRNA population in irradiated cells and, if this was not the case, how many miRNAs were sufficient to obtain the best correlation between predictions of our model and the experimental data. The contribution of particular miRNAs to modulation of the expressions of different mRNAs is reflected by the parameters *∆k*_*i*_, and to answer the question asked we proceeded according to the algorithm depicted in Fig. 6a. As before, Me45 cell line is used as reference, with results for the remaining cell lines detailed in the Supplement.

The first step was to consider miRNAs one-by-one, to calculate fold changes of mRNA expressions predicted from the *∆k*_*i*_ parameters, and to compare these to the experimental data. Calculations were repeated for each miRNA using Me45 cell data, and rank was ascribed to each miRNA based on the Spearman correlation coefficient between predicted and experimental results. The highest ranked 30 miRNAs with their parameters *∆k*_*i*_. and the corresponding Spearman correlation coefficients are shown in Table 3. Results for all miRNAs are presented in Additional file 5: Table S3 of Supplementary Material. Ranks reflect the predicted influence of particular miRNAs on changes of the global mRNA expression in irradiated cells.

**Table 3:** Highest ranking miRNAs (Me45 cells)

Rank	miRNA	Correlation coefficient *ρ*	Parameter *∆k*_*i*_.
1	hsa-miR-762	0.4067	− 0.00192
2	hsa-miR-638	0.3885	− 0.01268
3	hsa-miR-4281	0.3777	−0.00403
4	hsa-miR-3648	0.3763	−0.01113
5	hsa-miR-1247-5p	0.3718	−0.0154
6	hsa-miR-1207-5p	0.3709	−0.00183
7	hsa-miR-1469	0.3671	−0.02113
8	hsa-miR-663a	0.3656	0.00038
9	hsa-miR-1914-3p	0.3647	−0.00879
10	hsa-miR-2861	0.3612	−0.00248
11	hsa-miR-1915-3p	0.3386	−0.00143
12	hsa-miR-122-5p	0.3373	−0.0073
13	hsa-miR-1268a	0.3372	0.00116
14	hsa-miR-744-5p	0.3356	0.01103
15	hsa-miR-362-3p	0.3334	−0.01466
16	hsa-miR-3656	0.3276	0.00076
17	hsa-miR-3141	0.3123	−0.00158
18	hsa-miR-548f	0.3101	0.01264
19	hsa-miR-663b	0.3050	−0.00226
20	hsa-miR-874	0.3038	−0.00104
21	hsa-miR-1226-5p	0.3001	−0.01623
22	hsa-miR-3196	0.2900	−0.00271
23	hsa-miR-1470	0.2899	−0.00797
24	hsa-miR-1181	0.2893	−0.00358
25	hsa-miR-548a-5p	0.2769	0.0026
26	hsa-miR-320a	0.2761	0.00804
27	hsa-miR-4270	0.2716	−0.00057
28	hsa-miR-23a-5p	0.2715	−0.01881
29	hsa-miR-340-5p	0.2713	0.00919
30	hsa-miR-296-3p	0.2684	−0.02642

The value of parameter *∆k*_*i*_ (negative or positive) did not appear to be correlated with miRNA rank; for example, for the highest ranked miR-762, *∆k*_*i*_ is negative and its absolute value is one of the lowest. Twenty-two of the 30 top-ranked miRNAs are characterized by negative *∆k*_*i*_, indicating that irradiation of cells tends to cause a decrease of the expression of the mRNAs with which they interact. Correlations of the model calculated mRNA changes with only one miRNA assumed active using the experimental data are presented in Fig. 6b, where the x-axis shows the rank of the miRNA used in particular calculation and the y-axis the Spearman’s correlation coefficient for the predicted and experimental results. The names of the miRNAs considered are found in Additional file 5: Table S3.

We addressed the question of how many miRNAs are necessary to obtain the highest correlation between the predicted and experimental results. The number of miRNAs used in the model was increased by one in successive steps and the calculated mRNA expressions were compared to the microarray results (Fig. 6c, d). The y-axis shows the correlation coefficients and the x-axis the rank numbers of the miRNA which was added to calculations in the model to compare with the previous calculation with a lower miRNA number. Figure 6c shows the results when successive miRNAs were included in the calculations ordered by the decreasing rank. For example number 2 on the x-axis indicates that in the model two miRNAs, 1 and 2 from the rank table, were included, and number 3 that the three miRNAs numbers 1, 2, and 3 from the rank table were included. Thus the correlation coefficient corresponding to number 2 on the x-axis refers to the hsa-miR-762 and hsa-miR-638 miRNAs (numbers 1 and 2 in rank, Table 3). In the case of these two miRNAs the correlation coefficient was 0.44, and it increased with the number of miRNAs included (0.471 for 3 miRNAs, 0.483 for 4 miRNAs, 0.495 for 5 miRNAs and so forth). For more than 30 miRNAs the correlation coefficient reached a plateau value of about 0.56. These results show that it is possible to obtain the maximum correlation between the model and the experimental data by considering only about 30 highest ranking miRNAs. We observed similar cumulative miRNA effects in other cell types (Additional file 8: Figure S5).

To examine to what extent this effect of saturation depends on the ordering of miRNA, we reversed the order of miRNA addition starting from those with the lowest rank (implicitly, with the lowest impact on predictions) and then adding miRNAs with increasing ranks (Fig. 6c). In this case, the correlation between model predictions and experimental values increased with increased miRNA number but reached a maximum value only when all miRNAs were included, suggesting that the miRNAs with highest ranks exert the highest impact on mRNA expression.

### Structural features of miRNAs with the highest impact on mRNA expressions

To characterize the subgroup of 30 highest-ranked miRNAs, we compared different structural features of these miRNAs with all remaining miRNAs and performed Mann–Whitney U tests to assess the significance of the differences. Results for the features statistically significant in Me45 and other cells are summarized in Table 4; the results for other features are summarized in Additional file 5: Table S3).

**Table 4:** Properties of the 30 highest-ranked miRNAs compared with the group of all other miRNAs, with Me45 cells as reference

Features	Me45 Cells			Top rank vs. other miRNA *p*-value			
	Average over all miRNAs	Average over top ranked miRNAs	Average over other miRNAs	Me45	K562	HCT116^**+/+**^	HCT116^**−/−**^
Number of total targeted transcripts ^a^	3711.41	5523.86	3574.53	**1.56 × 10** ^**–10 b**^	**1.00 × 10** ^**–16 b**^	**3.20 × 10** ^**–09 b**^	**7.17 × 10** ^**–08 b**^
GC content in mature miRNA [%]	52.60	70.72	51.23	**7.95 × 10** ^**-09b**^	**3.26 × 10** ^**–11 b**^	**1.35 × 10** ^**–05 b**^	**0.001** ^**b**^
GC content in seed motif [%]	53.93	71.92	52.57	**1.93 × 10** ^**–07 b**^	**1.23 × 10** ^**–11 b**^	**8.37 × 10** ^**–05 b**^	**0.002** ^**b**^
GC content in pre-miRNA [%]	52.90	66.44	51.88	**2.55 × 10** ^**–07 b**^	**1.08 × 10** ^**–08 b**^	**3.69 × 10** ^**–05 b**^	**0.004** ^**b**^
Number of GC pairs in pre-miRNA	15.73	19.74	15.43	**0.027** ^**b**^	**0.001** ^**b**^	**0.005** ^**b**^	0.306
Number of unpaired fragments in mature double stranded miRNA	2.60	3.14	2.57	**0.019** ^**b**^	0.243	0.144	0.526
Length of complementary fragments in pre-miRNA	5.81	4.69	5.90	**0.012** ^**b**^	**0.042** ^**b**^	0.072	0.654
Length of mature miRNA	21.61	21.14	21.65	**0.031** ^**b**^	**0.028** ^**b**^	0.522	0.169
Parameter *∆k*_*i*_	0.00031	−0.0039	−0.000042	**0.022** ^**b**^	**0.016** ^**b**^	**0.047**	0.103
Spearman’s rho in ranking	0.12	0.33	0.10	**7.24 × 10** ^**–20 b**^	**2.48 × 10** ^**–20 b**^	**1.80 × 10** ^**–20 b**^	**4.73 × 10** ^**–20 b**^

^a^Average of values calculated for each single miRNA, based on targeted motifs obtained as described in Methods

^b^Boldface numbers indicate statistically significant differences

The influence of ionizing radiation on mRNA expressions might depend not only on structural features of transcripts, but also on features of miRNAs; for example, irradiation could influence interactions between miRNAs and other components of RISC complexes. We chose features potentially important for binding of pre-miRNA to Argonaute and/or other RISC proteins. These include pre-miRNA length, length of mature miRNA, nucleotide composition and hairpin length, and degree of complementarity. We also included structural features that might directly influence miRNA-mRNA interactions, such as seed nucleotide composition, and total number of targeted transcripts. We then compared the average numerical characteristics in the top-ranked and other miRNA groups (Table 4). All statistics were calculated using our custom tools and later used to compare the top ranked to all other miRNA groups based on their average values. The features were selected based on Me45 cell line results, using the *p*-value < 0.05 criterion. For completeness, results on the remaining cell lines were included in the Table 4.

The 30 highest-ranked miRNAs have a significantly higher content of G and C particularly in their seed motifs, they target a higher number of transcripts, and show a higher expression level than other miRNAs. In addition, the 30 highest-ranked miRNAs show features of the secondary structure of pre-miRNA such as length of complementary fragments (Table 4) and of the double-stranded form of mature miRNA, which suggest that the highest-ranked miRNAs differ from the other miRNAs also in this respect.

### Cellular processes influenced by the highest ranked miRNAs

We identified the KEGG pathways terms corresponding to genes with transcripts containing sequence motifs targeted by the 30 top-ranked miRNAs. Table 5 shows the example of pathways for Me45 cells. We used DIANA-mirPath [45] to find these pathways, but it was not possible to find detailed information about miRNAs which have been discovered only recently. The top-ranked miRNAs target mRNAs in 35 KEGG pathways (*p*-value < 0.05). Examples of these pathways are shown in Table 5 and all are presented in the (Additional file 9: Table S4).

**Table 5:** Selected pathways regulated by the 30 highest-ranked miRNAs

KEGG pathway	*p*-value	Number of genes	Number of miRNAs
Hippo signaling pathway	0.00003	67	12
Transcriptional misregulation in cancer	0.0003	82	14
Circadian rhythm	0.0022	20	7
Endocytosis	0.0033	93	14
ErbB signaling pathway	0.0048	43	13
TGF-beta signaling pathway	0.0117	38	12
mTOR signaling pathway	0.0117	32	13
Wnt signaling pathway	0.0119	65	13
Hedgehog signaling pathway	0.0242	27	13
FoxO signaling pathway	0.0270	60	10

Many of these pathways are connected with cellular signalling and responses in different diseases. Identified pathways are consistent with top-rank miRNAs being connected with irradiation response. For example, the *Hippo signaling pathway* (*p*-value 3.65 × 10^−5^ , 67 genes, 12 miRNAs) is involved in the responses to cellular stresses, including mechanical stress, DNA damage, and oxidative stress, aimed at maintaining homeostasis at the cellular and organic levels [46]. In addition, *FoxO signalling pathway* (*p*-value 0.027, 60 genes, 10 miRNAs) is important in the activation of the ATM pathway and the maintenance of genome integrity in response to DNA damage [47]. Finally, *mTOR signalling pathway* (*p*-value 0.011, 32 genes, 13 miRNAs) is responsible for redox homeostasis and radiosensitivity [48].

### Features of mRNAs which better fit the model to indicate miRNA regulation

To search for differences between mRNAs that fit and those which do not fit our model, based on the Me45 cell line, we compared a subset of structural features of mRNAs with expression changes that could or could not be predicted and of their genes. Features considered include the numbers of transcription factor binding sites in promoter regions, numbers of ARE motifs of various types in the 3’-UTRs of their primary transcripts, nucleotide composition (percentage of G and C), and mRNA turnover rates. We divided all mRNAs into two groups based on how well their expression level changes are predicted by the model. The threshold has been defined in the terms of the difference between predicted and observed log_2_ fold change, at the arbitrary value of 0.5. We then compared the structural features of the mRNAs in these groups using a two sample *t*-test, which allowed us to identify the discriminating factors summarized in Table 6. The group of mRNAs whose levels change as predicted by the model tends to have longer 3’ UTRs, fewer AU-rich regions, less MYC response elements in their promoters, and shorter turnover times.

**Table 6:** Features characterizing mRNAs with good or poor fit to the model, with Me45 cells as reference

Feature	Me45 Cells: Average over		Good vs. poor fit *p*-value			
	Good fit	Poor fit	Me45	K562	HCT116^+/+^	HCT116^−/−^
3’-UTR average length [nucleotides]	1466.3	1229.2	**7.23 × 10** ^**–06** b^	**0.007** ^b^	0.106	0.117
Average turnover time [h]	7.912	8.766	**0.0030** ^b^	**0.027** ^b^	0. 609	0.829
Average number of ARE consensus motifs/gene	0.217	0.263	**0.0291** ^b^	0.791	**7.19 × 10** ^**–05** b^	**0.004** ^b^
MYC response elements / gene	1.434	1.561	**0.0004** ^b^	**0.003** ^b^	0.079	**0.030** ^b^

^b^Boldface numbers indicate statistically significant differences

The criteria for mRNA classification used in the calculations in Table 6 were chosen arbitrarily. To examine the influence of the classification method on the results, we calculated the differences while varying the cut-off values with a 0.1 interval. A higher value of the cut-off criteria resulted in a lower number of mRNAs that showed a good fit to the data. By increasing the cut-off value, we found increasing structural differences between mRNAs and genes in groups that showed good or poor fit. mRNAs that show a good agreement with the model were characterized by a lower number of transcription factor binding sites and longer 3′-ends (Additional file 10: Figure S6). This is consistent with regulation of some classes of mRNAs being more strongly influenced by transcription factors than by miRNAs.

### The influence of miRNA-mRNA interactions on response to radiation is cell type specific and depends on the radiation dose and time from irradiation

In addition to the short-term 1 h time point we also explored, in less detail, the 12 and 24 h time points. The correlation coefficient between transcriptome changes predicted by the model and experimentally assayed changed in time suggesting that in Me45 cells the influence of miRNA-mRNA interactions on transcriptome changes decreased with time (Table 7).

**Table 7:** The correlation coefficients (*ρ*) of model predicted and experimentally assayed transcriptome expressions change in time. Me45, K562 and HCT116 cells were irradiated with 2Gy of ionizing radiation, the levels of transcripts were assayed at different time points after irradiation in microarray experiment and predicted by model simulations

Cell lines	Correlation coefficient (ρ)		
	1 h	12 h	24 h
Me45	0.612	0.550	0.336
K562	0.476	0.356	0.414
HCT116^+/+^	0.587	0.645	0.688
HCT116^−/−^	0.636	0.547	0.586

Correlation coefficients between predicted and experimentally assayed mRNA expressions decreased with time in Me45 cells, which suggests that in this cell line these interactions had the highest influence on transcriptome changes directly after irradiation, and later the influence of other mechanisms increased. However for other cell types, the correlation coefficients of predictions versus the experimental results were not changing monotonously, and the patterns were cell type specific (see Table 7).

In addition, we performed analysis of experiments similar to ours, involving a set of cell types assayed at different times after irradiation. The datasets are available in the ArrayExpress database; some of their characteristics are listed in Additional file 1: Table S1. The correlation coefficients between experiment and our model prediction are always less than 1 since factors different from miRNAs influence the mRNA expressions and reduce the correlation. The correlation coefficients may be treated as a measure of the role of miRNA in regulation of mRNA expressions after action of stressing factor. Irradiated cells for which we performed model simulations differed with respect to correlation coefficient values. Accordingly, for estimation of the impact of miRNA-mRNA interactions on the response to radiation we used the threshold for good and poor fit classification based on the correlation coefficient greater or less than 0.2. As seen in Additional file 3: Table S2, some results do not pass this criterion suggesting that transcriptome changes in these experiments are not connected to changes of miRNA-mRNA interaction change.

**Fig. 7:**
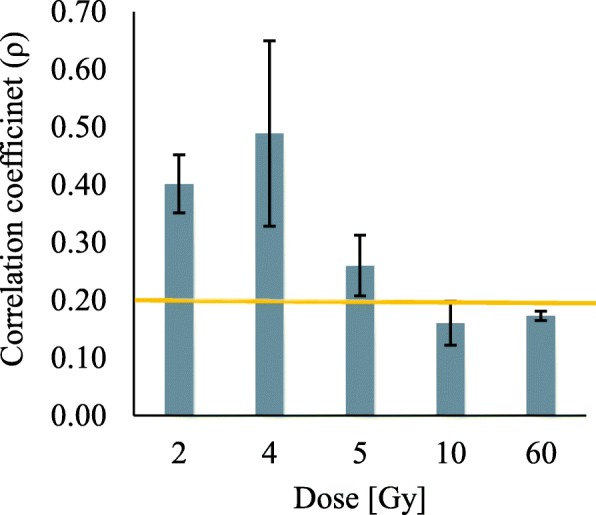
Summary of the study of model performance using publicly available miRNA and mRNA datasets. Values of correlation coefficient (ρ) calculated for experimental and simulation data depend on irradiation dose. Bars represent mean values with minimum and maximum values calculated based correspondingly on 3 (2 Gy), 14 (4 Gy), 2 (5 Gy), 4 (10 Gy) and 3 (60 Gy) datasets

Values of the correlation coefficient (*ρ*) have been calculated for experimental and simulation data for each irradiation dose. A summary of results is depicted in Fig. 7. Bars represent mean values with minimal and maximal values calculated based correspondingly on 3 datasets with 2 Gy radiation dose, 14 with 4 Gy, 2 with 5 Gy, 4 with 10 Gy, and 3 with 60 Gy. Comparisons of predicted and experimental results for data available in the ArrayExpress database, involving cells irradiated with different doses, showed that a good fit between model and experiment is only achieved for doses lower than 5Gy. Concluding, it seems that the regulation of mRNA expressions in irradiated cells depends on miRNA only if the direct effects of irradiation dominate cell response.

## Discussion

As it is known, one major effect of ionizing radiation on cells is the induction of a massive wave of reactive oxygen species (ROS) which precedes the changes in mRNA expressions by a few minutes [30]. The results reported here are consistent with the hypothesis that these rapid changes in mRNA expressions result, at least in part, from damage to miRNAs and mRNAs by ROS which leads to perturbation of their interactions. It is not generally recognized that RNA is even more sensitive than DNA to oxidative damage [49]; the effects of ROS on RNA have been less explored, but in HeLa cells exposed to sub-milimolar concentrations of H_2_O_2_ for 1 h, they led to a 50% decrease in the level of 8-oxoG in total RNA (reviewed in [50]). In our top-ranked miRNAs which seem to influence mRNA expressions most, the seed region is significantly enriched in GC; G has the lowest ionizing potential of the nucleic acid bases and G-specific damage by ROS is well documented for DNA and is therefore likely for RNA [51]. mRNAs whose primary transcripts contain a longer than average CUG repeat in their 3’UTR might be more sensitive to down-regulation by miRNAs which bind to them, as observed for regulation of the myotonic dystrophy protein kinase (DMPK) gene by miR-15b/16 where an increased number of CUG repeats increases the efficiency of down-regulation [52]. Our model summarizes the effects of all available miRNAs on each particular mRNA, assuming that the effects depend on the number of different miRNA-targeted sequences and the miRNA concentrations. Some transcripts contain a large number of possible miRNA binding sites; for example, the MDM2 and MDM4 transcripts have 760 and 1276 potential miRNA binding sites, respectively [53]. How many of these sites are active, seems unclear.

It is notable that a specific group of thirty miRNAs is predicted to be mainly responsible for the observed miRNA-dependent mRNA changes in irradiated cells. These are the miRNAs sufficient for obtaining a maximal correlation between the predicted and experimental results. Fifteen of the 30 top-ranked miRNAs participate in cellular processes involved in cancer development and metastasis. The one highest-ranked in Me45 cells, miRNA-762, promotes breast cancer cell proliferation and invasion [54] and the second, miR-638, plays a role in embryonic development and tissue differentiation [55–58] and is involved in the development of numerous types of tumors including gastric cancer [59–62], colorectal carcinoma [63, 64], non-small cell lung cancer [65, 66], basal cell carcinoma [67], nasopharyngeal carcinoma [68], and melanoma [69]. Expressions of the high-ranked miRNAs 3648, 663a and b, 548 t, miR-1207, 1225-5p, 3141, and 4270 (Table 3) are correlated with breast cancer recurrence and prostate cancer metastasis [70, 71].

We note that our model has some limitations. First, it considers only one of several regulatory mechanisms that could be perturbed by radiation, and therefore it is not expected to reproduce all changes in mRNA expressions. mRNA expressions depend not only on interactions with miRNAs but also on their transcription rate and half-lives, both of which could be affected by radiation. Other more complex processes which are not considered in our model could potentially contribute to the response of the level of a mRNA to irradiation, including effects on only some of the miRNA-transcript interactions which regulate its level [52] or opposing effects which result in no change in level. Transcripts which contain targets for miRNAs separated by 7–40 bases and therefore with a greater possible cooperativity may be more sensitive to down-regulation, as observed for the effects of miR-148a and miR-206 on mRNA transcribed from the DMPK gene [52, 72]. Further examples of increased sensitivity to down-regulation include decoy-based relief of mRNA repression by miRNA [72, 73], competitive endogenous RNAs (ceRNAs) which bind miRNAs, specific pseudogenes which relieve miRNA-based repression of mRNAs [72, 74–78], or the influence of AU-rich binding proteins such as HuR [72, 73]. These exceptions, which are uncommon relative to the miRNA binding considered in our model, do not seriously affect the validity of our modelling approach.

The second limitation is that only degradation of mRNAs can be detected by microarrays but not inhibition of mRNA translation, and in no case we do obtain a full correlation between the fold changes of mRNA expressions predicted by the model and data from microarrays. Nevertheless, the changes of mRNA expressions predicted by this model show high correlation coefficients (ca. 0.6) with experimental data and are statistically significant. Our modeling approach thus appears to reflect real relationships which occur in vivo, and suggests that a part of the ionizing radiation-induced changes of mRNA expressions depends on perturbed miRNA-mRNA interactions. This modeling approach could be used to identify miRNAs which participate in responses of cells to other environmental challenges.

## Additional files

Additional file 1: **Table S1.** Summary of dataset at our disposal downloaded from ArrayExpress database. Table includes information about tissue and cell names, experimental settings and microarray platform and ID numbers of individual experiments. (XLSX 10 kb)

Additional file 2: **Figure S1.** Model prediction (correlation coefficient value between experimental and simulation data ρ) for different noise level thresholds for mRNA in microarray data. Three thresholds were tested 4 (~ 1700 mRNAs), 6 (~ 1000 mRNAs) and 8 (~ 500 mRNAs), as described in Measurement of mRNA and miRNA levels section. (TIF 80 kb)

Additional file 3: **Table S2.** Summary of each dataset (number of expressed mRNAs and miRNAs), experimental conditions (radiation dose in Gy, time of observation) and results of model simulations and validation described as Spearman correlation coefficient values for all analysed cell lines. (XLSX 14 kb)

Additional file 4: **Figure S2.** Three methods for *C* matrix validation. A) Each value in *C* matrix is represented by random number. Numbers were drawn from uniform distribution [0–1]. Resulting *C* matrix was used to simulate predicted mRNA fold change. Example of single prediction is presented on the left in the form of scatter plot, a result of thousand predictions is presented on the right in the form of histogram, where *x* axes indicated *ρ* returned by model. B) The *C* matrix was permuted (the values change the initial location). Resulting *C* matrix was used to simulate predicted fold change. Example of single prediction is presented on the left in the form of scatter plot, a result of thousand predictions is presented on the right in the form of histogram, where *x* axes indicated ρ returned by model. C) Original *C* matrix was used to estimate model parameters, after that the *C* matrix was permuted and used for prediction of mRNA changes. Example of single prediction is presented on the left in the form of scatter plot, a result of thousand predictions is presented on the right in the form of histogram, where *x* axes indicated *ρ* returned by model. (TIF 1058 kb)

Additional file 5: **Table S3.** The calculated values for all miRNAs expressed in Me45, K562, HCT116+/+ and HCT116−/− cells together with other characteristics of the miRNAs studied. (XLSX 345 kb)

Additional file 6: **Figure S3.** Proportion of genes represented by one or more transcripts in four cell lines. (TIF 561 kb)

Additional file 7: **Figure S4.** Correlations between the predicted and the experimentally observed fold changes of mRNA levels in all available cell lines. A) AG1522 cell line, 3 h after radiation, dose 2 Gy, ρ = 0.378. B) AG1522cell line, 3 h after radiation, dose 5 Gy, ρ = 0.222. C) MOLT4 (Bay) cell line, 2 h after radiation, dose 4 Gy, ρ = 0.215. D) MOLT4 (DMSO) cell line, 2 h after radiation, dose 4 Gy, ρ = 0.196. E) DU145cell line, 2 h after radiation, dose 10 Gy, ρ = 0.172. F) HCAEC (SD) cell line, 6 h after radiation, dose 10 Gy,, ρ = 0.108. G) HCAECs (MF) cell line, 6 h after radiation, dose 10 Gy, ρ = 0.198. H) MOLT4 cell line, 2 h after radiation, dose 5 Gy, ρ = 0.297. I) PBMC cell line, 2 h after radiation, dose 60 Gy, ρ = 0.179. J) PBMC cell line, 4 h after radiation, dose 60 Gy, ρ = 0.163. K) PBMC cell line, 20 h after radiation, dose 60 Gy, ρ = 0.175. L) SC3 cell line, 2 h after radiation, dose 10 Gy, ρ = 0.160. M) WI38 cell line, 1 h after radiation, dose 2 Gy, ρ = 0.366. N) WI39 cell line, 2 h after radiation, dose 2 Gy, ρ = 0.459 (TIF 812 kb)

Additional file 8: **Figure S5.** Influence of individual miRNAs on the prediction of radiation-induced changes of mRNA levels in K562 (A, B, C) HCT116+/+ (D, E, F) and HCT116−/− (G, H, I) cells. (A, D, G) Ranking miRNA according to correlation coefficient to lowest. (B, E, H) Using an increasing number of miRNAs added according to decreasing rank. (C, F, I) Using an increasing number of miRNAs added according to increasing rank. (TIF 878 kb)

Additional file 9: **Table S4.** KEGG pathways identified for 30 top-rank miRNAs in Me45 cell line. (XLSX 12 kb)

Additional file 10: **Figure S6.** Influence of the classification criteria on differences between mRNAs with good or poor fit to the model. The plot is an extended version of the data in Table 3, showing the same features but using variable fit error cutoffs for classification based on how well they fit the model. (TIF 182 kb)

## Abbreviations

ceRNA: Competitive endogenous RNA
EM: Expectation–maximization
FC: Fold change
HCT116^−/−^: HCT116 p53 −/− cell line
HCT116^+/+^: HCT116 p53+/+ cell line
K562: Human immortalised myelogenous leukemia cell line
Me45: Human melanoma cell line (established in the Center of Oncology Gliwice)
miRNA: MicroRNA
mRNA: Messenger RNA
RISC: RNA-induced silencing complex
TF: Transcription factor
