# Supplementary figures and images for: A mathematical model as a tool to identify microRNAs with highest impact on transcriptome changes
Source: BMC Genomics. 2019 Feb 6;20:114. doi: 10.1186/s12864-019-5464-0 (PMC6366035; doi:10.1186/s12864-019-5464-0)

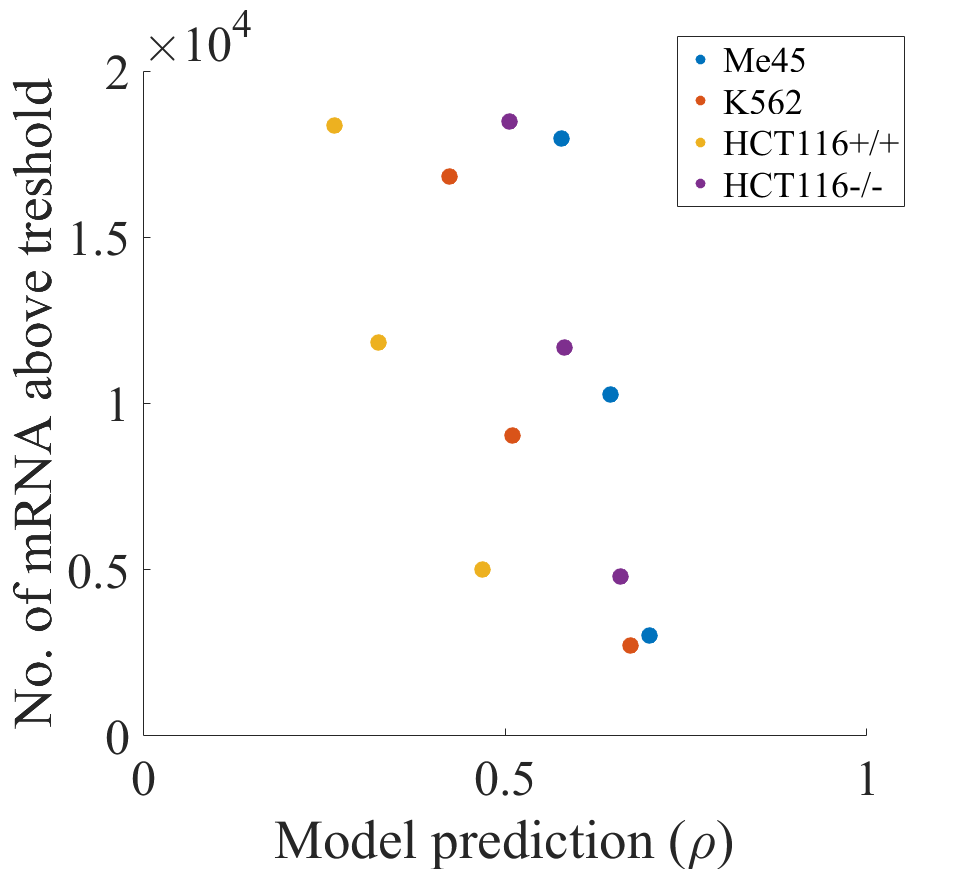

Supplement: Supplementary file 2 — Figure S1. Model prediction (correlation coefficient value between experimental and simulation data ρ) for different noise level thresholds for mRNA in microarray data. Three thresholds were tested 4 (~ 1700 mRNAs), 6 (~ 1000 mRNAs) and 8 (~ 500 mRNAs), as described in Measurement of mRNA and miRNA levels section. (TIF 80 kb) [file 12864_2019_5464_MOESM2_ESM.tif]

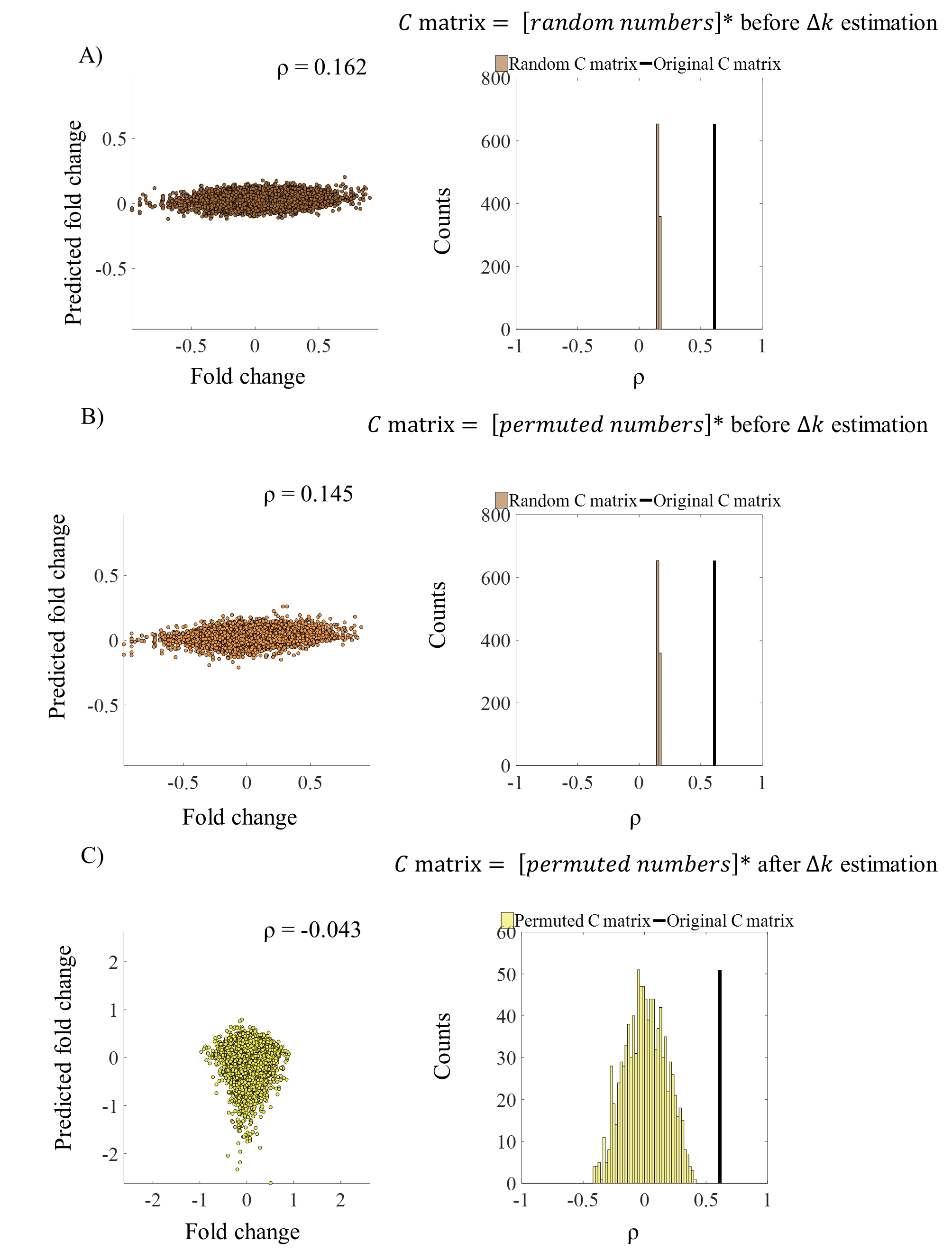

Supplement: Supplementary file 4 — Figure S2. Three methods for C matrix validation. A) Each value in C matrix is represented by random number. Numbers were drawn from uniform distribution [0–1]. Resulting C matrix was used to simulate predicted mRNA fold change. Example of single prediction is presented on the left in the form of scatter plot, a result of thousand predictions is presented on the right in the form of histogram, where x axes indicated ρ returned by model. B) The C matrix was permuted (the values change the initial location). Resulting C matrix was used to simulate predicted fold change. Example of single prediction is presented on the left in the form of scatter plot, a result of thousand predictions is presented on the right in the form of histogram, where x axes indicated ρ returned by model. C) Original C matrix was used to estimate model parameters, after that the C matrix was permuted and used for prediction of mRNA changes. Example of single prediction is presented on the left in the form of scatter plot, a result of thousand predictions is presented on the right in the form of histogram, where x axes indicated ρ returned by model. (TIF 1058 kb) [file 12864_2019_5464_MOESM4_ESM.tif]

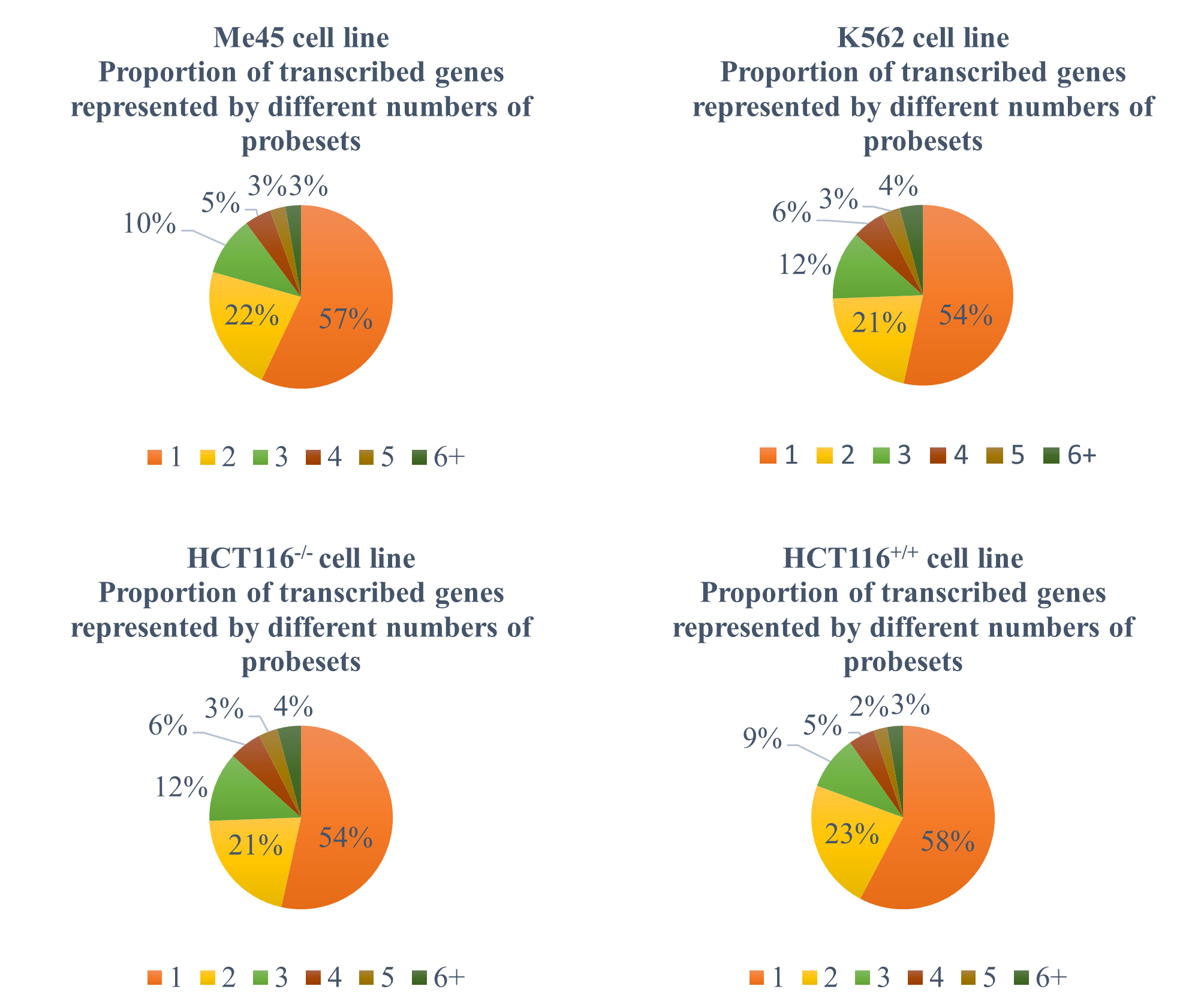

Supplement: Supplementary file 6 — Figure S3. Proportion of genes represented by one or more transcripts in four cell lines. (TIF 561 kb) [file 12864_2019_5464_MOESM6_ESM.tif]

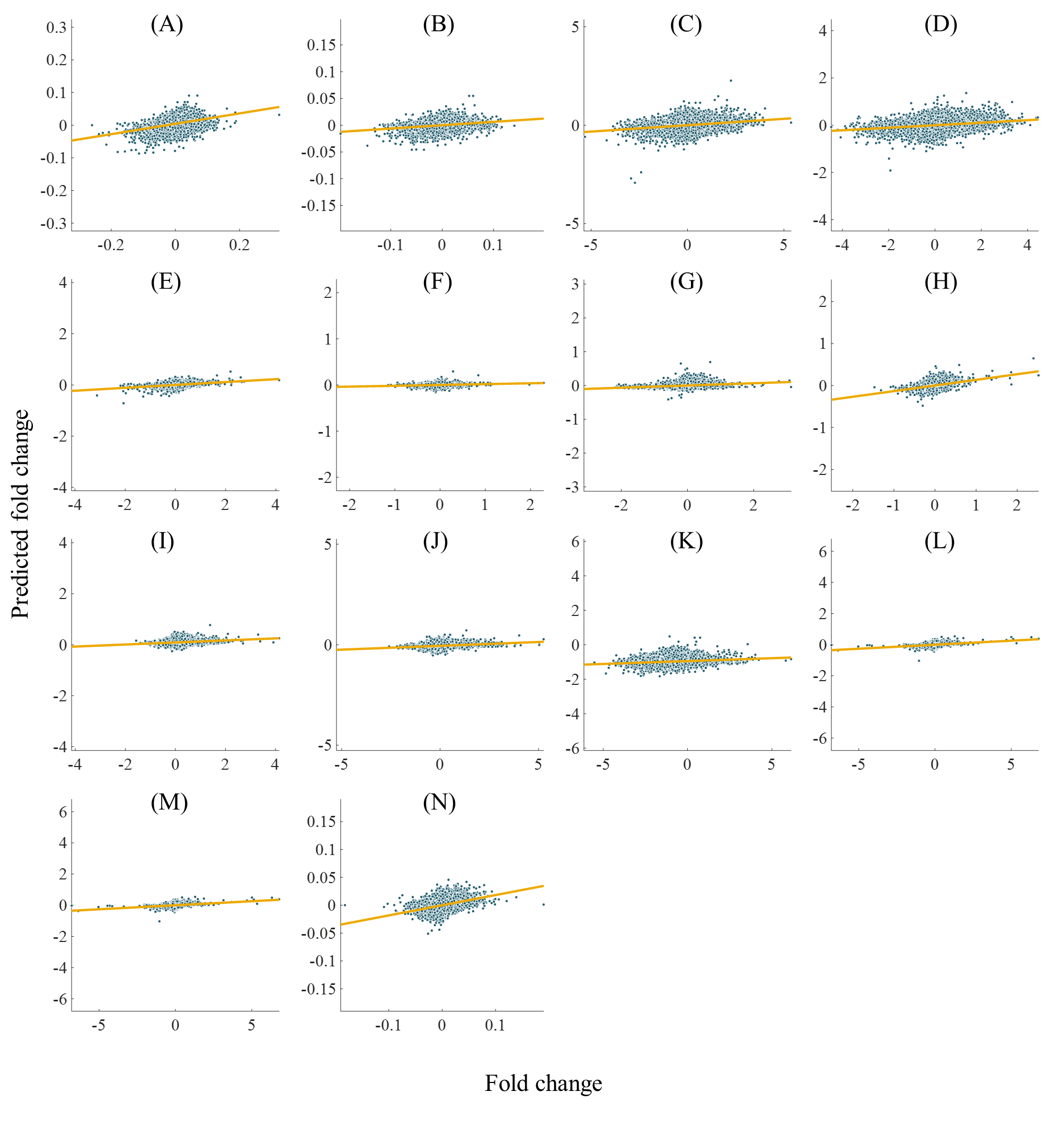

Supplement: Supplementary file 7 — Figure S4. Correlations between the predicted and the experimentally observed fold changes of mRNA levels in all available cell lines. A) AG1522 cell line, 3 h after radiation, dose 2 Gy, ρ = 0.378. B) AG1522cell line, 3 h after radiation, dose 5 Gy, ρ = 0.222. C) MOLT4 (Bay) cell line, 2 h after radiation, dose 4 Gy, ρ = 0.215. D) MOLT4 (DMSO) cell line, 2 h after radiation, dose 4 Gy, ρ = 0.196. E) DU145cell line, 2 h after radiation, dose 10 Gy, ρ = 0.172. F) HCAEC (SD) cell line, 6 h after radiation, dose 10 Gy,, ρ = 0.108. G) HCAECs (MF) cell line, 6 h after radiation, dose 10 Gy, ρ = 0.198. H) MOLT4 cell line, 2 h after radiation, dose 5 Gy, ρ = 0.297. I) PBMC cell line, 2 h after radiation, dose 60 Gy, ρ = 0.179. J) PBMC cell line, 4 h after radiation, dose 60 Gy, ρ = 0.163. K) PBMC cell line, 20 h after radiation, dose 60 Gy, ρ = 0.175. L) SC3 cell line, 2 h after radiation, dose 10 Gy, ρ = 0.160. M) WI38 cell line, 1 h after radiation, dose 2 Gy, ρ = 0.366. N) WI39 cell line, 2 h after radiation, dose 2 Gy, ρ = 0.459 (TIF 812 kb) [file 12864_2019_5464_MOESM7_ESM.tif]

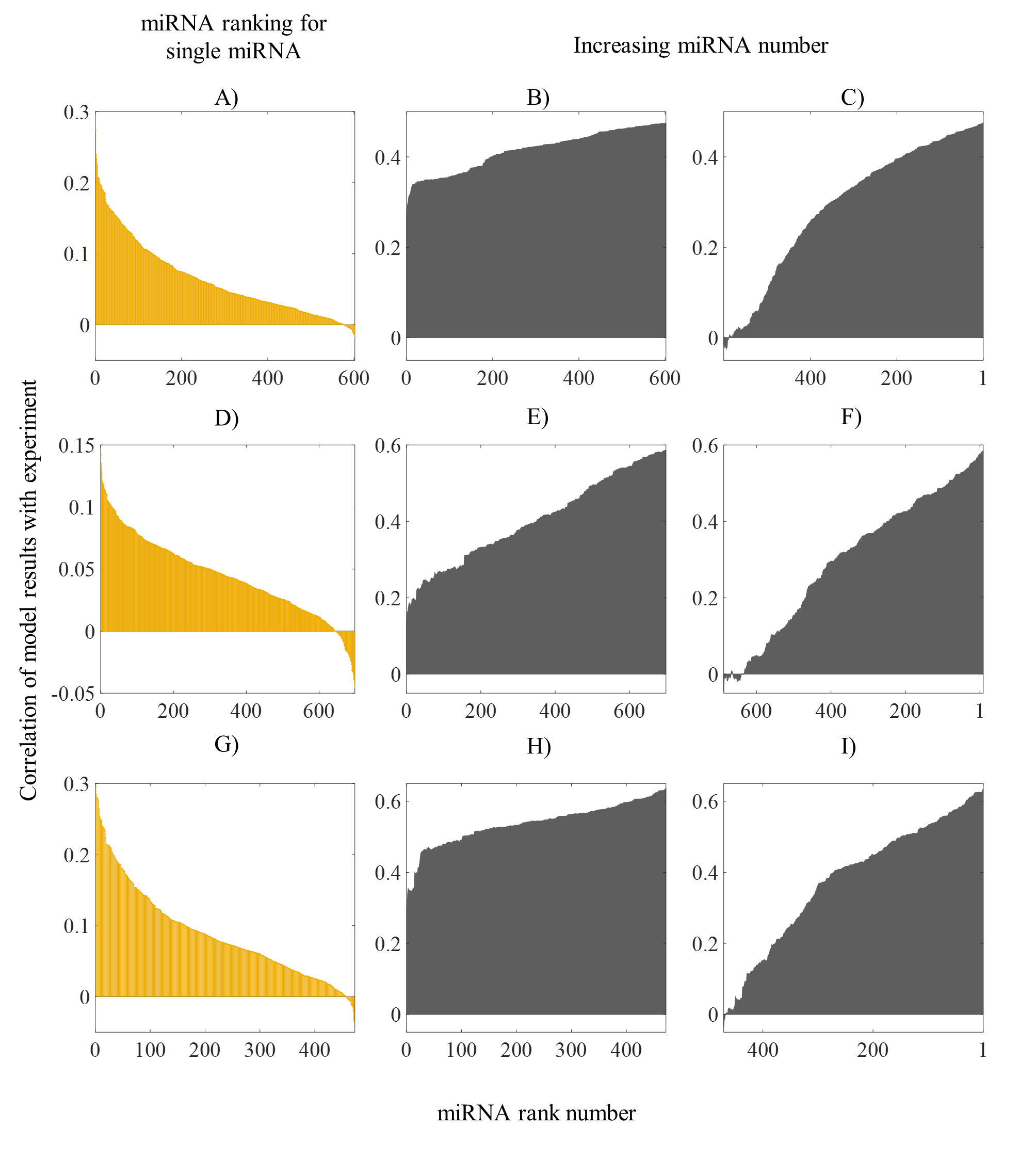

Supplement: Supplementary file 8 — Figure S5. Influence of individual miRNAs on the prediction of radiation-induced changes of mRNA levels in K562 (A, B, C) HCT116+/+ (D, E, F) and HCT116−/− (G, H, I) cells. (A, D, G) Ranking miRNA according to correlation coefficient to lowest. (B, E, H) Using an increasing number of miRNAs added according to decreasing rank. (C, F, I) Using an increasing number of miRNAs added according to increasing rank. (TIF 878 kb) [file 12864_2019_5464_MOESM8_ESM.tif]

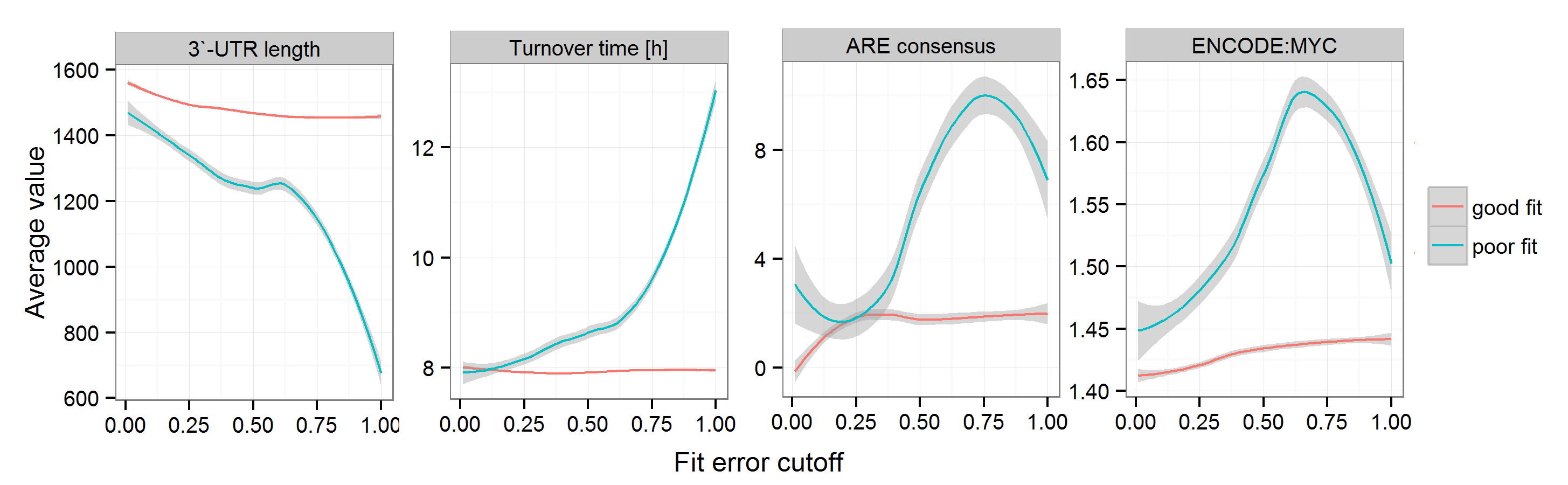

Supplement: Supplementary file 10 — Figure S6. Influence of the classification criteria on differences between mRNAs with good or poor fit to the model. The plot is an extended version of the data in Table 3, showing the same features but using variable fit error cutoffs for classification based on how well they fit the model. (TIF 182 kb) [file 12864_2019_5464_MOESM10_ESM.tif]
